# Medical Erudition

**Published:** 1868-03-15

**Authors:** 


### MEDICAL ERUDITION.

February 14th, 1868.

Dr. Allen:—The following is sent to you as a sample of the *erudition* of some of our compeers in the "theory and practice of medicine." The foregoing is a true *copy* "verbatim et literatim et punctuatim," of advice and prescription given to a person of this city by one, J. Dodge, who calls himself Doctor, (should be "*Doctus.*")

Chas. M. Clark, M.D.

Sylvester green Co Wis. Feb the 10 1868

Barbra Kiblen your Lungs the Stronger they fill better your Liver is More Active the Digestive organs Do their office Work More freely The Stomach is Stronger the Stomach and Nerves Dont

Affect your head As much the Nervous system is Stronger the Uterin organs the Stronger.

Prescription take two thirds of A table Spoonful of the Columbo. bitters a half hour before breakfast for A Syrup one oz of Lifeeverlasting one oz of Yellow Dock Root one fourth of A oz Seneca Snake Root one fourth of A oz of White Pond Lily Root Put All of these in six quarts of boiling Water Simmer it to one quart then strain it & sweeten it With Loaf Sugar to take Add half A Pint of fourth Proof brandy take one table spoonful A half hour before before Dinner & Supper the White of one hens egg onetable spoonfull of Loaf Sugar one table spoonful of fourth Proof brandy beat All Well together & take this quantity in the Middle of the Afternoon Drink Scull cup Tea With your Meales Make it Weak not not boil eat Light food Rub the orguinum Liniment on your forehead & temples, Morning noon and night, & smell of it the same time. Prepare the Linament As follows. one oz oil of oniganum half oz of Sweet oil half oz of Spiritz harts horn half oz of Laudanum Put All these together and apply it as Directed.

Prepare the columbo bitters As follows. one oz of columbo Root half oz of Rhubarb, half oz of extract Dandelion one fourth of A oz of Aloes. Put all of them in one quart of Whiskey & take it As Directed Continue these Medecines seven Weeks, then you are to be seen again.

examination & Prescription by Dr J. Dodge.

